# Supplementary material for: Spectral and Redox Properties of a Recombinant Mouse Cytochrome b561 Protein Suggest Transmembrane Electron Transfer Function
Source: Molecules. 2023 Feb 28;28(5):2261. doi: 10.3390/molecules28052261 (PMC10005133; doi:10.3390/molecules28052261)
Supplement: Supplementary file 1 [file molecules-28-02261-s001.zip › molecules-2227752-supplementary.pdf]

Supplementary material

Spectral and redox properties of a recombinant mouse cytochrome *b*561 protein suggest transmembrane electron transfer function

Alajos Bérczi [1], Zsuzsanna Márton [1], Krisztina Laskay [1], András Tóth [1,2], Gábor Rákhely [1,2], Ágnes Duzs [1,2], Krisztina Sebők-Nagy [1], Tibor Páli [1], and László Zimányi [1,*]

[1] Institute of Biophysics, Biological Research Centre Szeged, Szeged, Hungary;
[2] Department of Biotechnology, University of Szeged, Szeged, Hungary
[*] Correspondence: zimanyi.laszlo@brc.hu

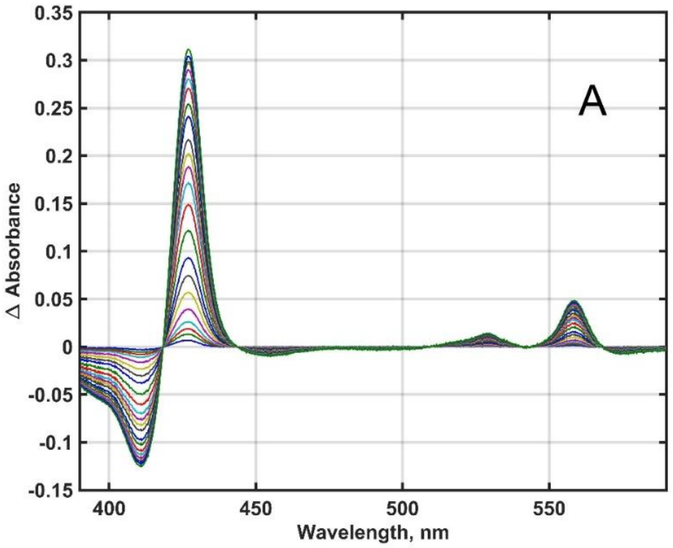

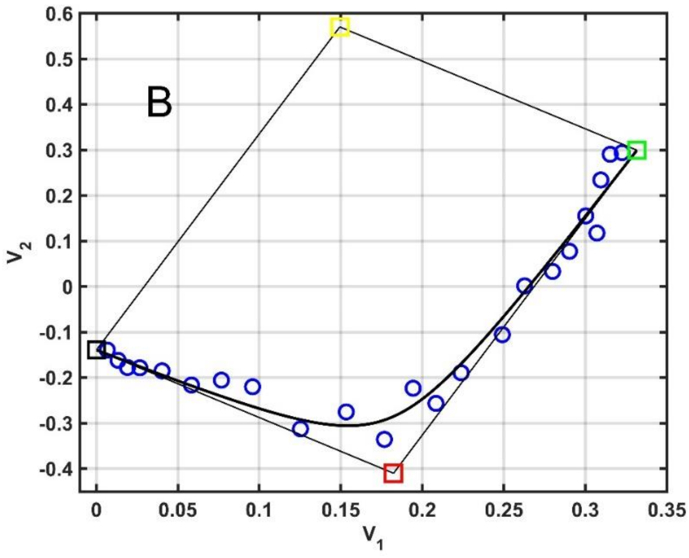

Figure S1. A: Difference spectra (**D** matrix) obtained from Figure 3A (main text) by subtracting the spectrum of the fully oxidized sample from each consecutive spectrum. B: 2D plot of the two significant titration eigenvectors in the **V** matrix (circles) obtained from the SVD analysis of the difference spectral data matrix. Thick line is the fit to Eq. 2. Squares correspond to the pure states as [ox/ox]: black; [red/ox]: red; [ox/red]: yellow; [red/red]: green.

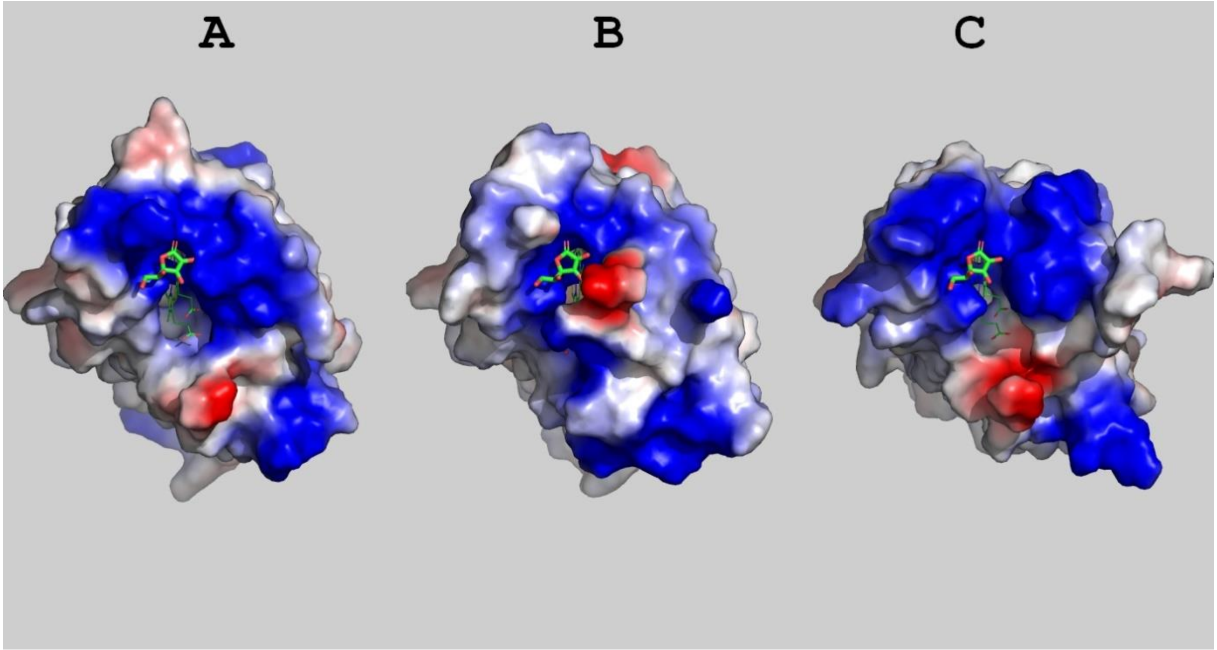

Figure S2. Experimental crystal structures of the *Arabidopsis thaliana* CYB561B2 (A) and the human duodenal CYB561A2 (B), as well as the homology modelled structure of Mm_CYB561D1 (C). View from the cytoplasmic side, colored according to the surface potential. The ASC ligands are shown as thick sticks and the cytoplasmic hemes as thin sticks.

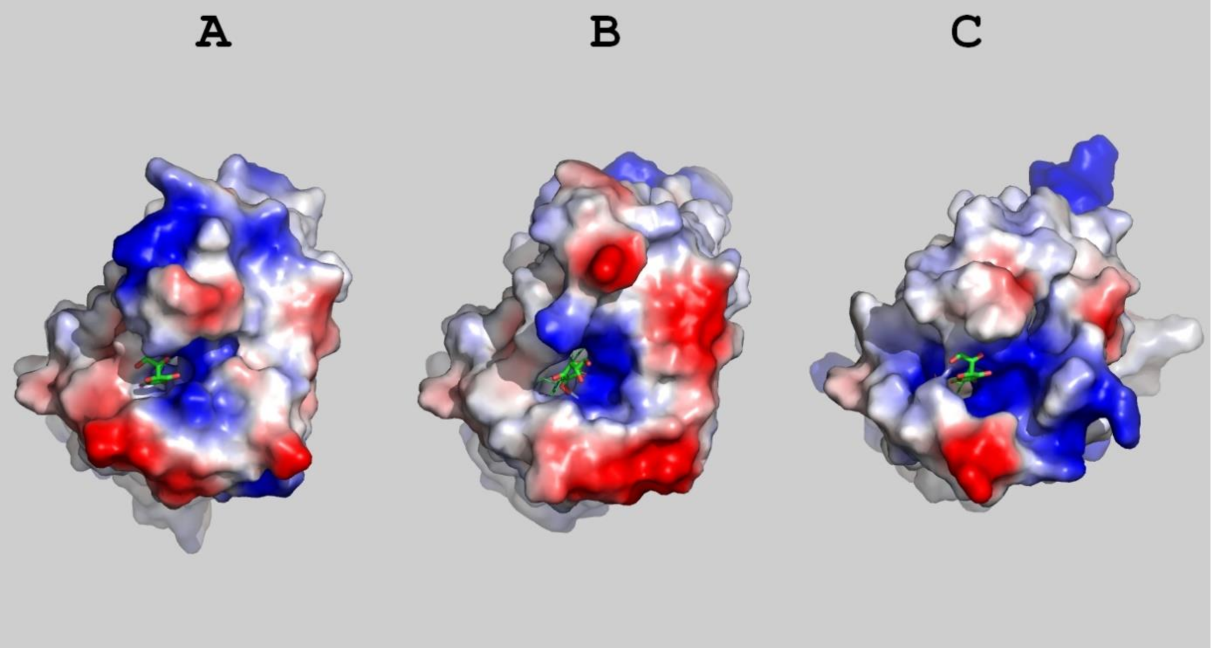

Figure S3. Experimental crystal structures of the *Arabidopsis thaliana* CYB561B2 (A) and the human duodenal CYB561A2 (B), as well as the homology modelled structure of Mm_CYB561D1 (C). View from the non-cytoplasmic side, colored according to the surface potential. The ASC ligands are shown as thick sticks and the non-cytoplasmic hemes (barely visible) as thin sticks.

Table S1. Solubilization efficacy of ascorbate-reducible (AscRed) Mm_CYB561D1 by different agents. Repetitions of independent solubilizations are in parentheses. Solubilization with detergents occured in 50 mM phosphate buffer (pH 7.0) and in the presence of ~1 mg/ml protein, 2:1 (w/w) ratio of detergents. For solubilization with SMA(2:1), the copolymer concentration was 2% (w/v) and 200 mM NaCl was also present in the 50 mM phosphate buffer (pH 7.8).

Solubilization efficacy of AscRed Mm_CYB561D1

Agent used	Short name used	c.m.c.	Percentage solubilized protein	Percentage solubilized AscRed Mm_CYB561D1	Fold purification
Dodecyl-β-D-maltoside (4)	DDM	0.1-0.4*	47.5	69.2	1.45
Triton X-100 reduced (2)	TX-100RS	0.24	26.1	15.1	0.58
Octaethylene glycol dodecylether (2)	$C_{12}E_8$	0.11	47.5	39.7	0.84
N-Tetradecyl-N,N-dimethyl-3-Ammonio-1-propanesulfonate (2)	SB3-14	0.1-0.4*	48.8	13.7	0.28
Styrene-maleic acid copolymer (2:1) (4)	SMA(2:1)	--	46.3	14.1	0.30

* - c.m.c. depends on the ionic strength of the medium at pH 7.0
